# Adult pancreatic hemangioma: a rare case report and literature review

**DOI:** 10.1186/s12893-020-00779-8

**Published:** 2020-06-03

**Authors:** Chong Jin, Jing-gang Mo, Hao Jiang, Lie-zhi Wang, Heng Zou, Kun-Peng Wang

**Affiliations:** 1 Department of General Surgery, Taizhou Central Hospital, Taizhou University Hospital, Taizhou, Zhejiang, 318000 China; 2 Department of General Surgery, Second Xiangya Hospital, Central South University, Changsha, 410000 Hunan China

**Keywords:** Pancreatic hemangioma, Computed tomography, Endoscopic ultrasonography, CD34

## Abstract

**Background:**

Adult pancreatic hemangioma is an extremely rare type of benign vascular tumor. To date, about 20 cases have been reported in the English literature. Adult patients with pancreatic hemangiomas usually have no specific symptoms, particularly in early stages. Therefore, it is difficult to detect and diagnose these lesions, which usually are identified during cross sectional imaging for an apparently unrelated causes or when biliary obstruction occurs because of compression by a tumor.

**Case presentation:**

This study presents the case of a 52-year-old female with a chief complaint of epigastric pain. Contrast-enhanced computed tomography revealed a well-defined mass with mildly inhomogeneous enhancement in the body of the pancreas. Endoscopic ultrasonography showed calcifications in the lesion, and a few small vessels were detected by Doppler imaging. The patient received a central pancreatectomy, and pathological examination confirmed the diagnosis of pancreatic hemangioma.

**Conclusion:**

In this report, we reviewed the clinical manifestations, radiologic features, preoperative diagnosis, pathologic characteristics, and surgical treatment of adult pancreatic hemangioma.

## Background

Adult pancreatic hemangioma is an extremely rare type of benign vascular tumor. To date, about 20 cases have been reported in the English literature [1]. Adult patients with pancreatic hemangiomas usually have no specific symptoms, particularly in early stages. Therefore, it is difficult to detect and diagnose these lesions, which usually are identified during cross sectional imaging for an apparently unrelated causes or when biliary obstruction occurs because of compression by a tumor. Here, we present the case of a 52-year-old female who underwent a central pancreatectomy for a hemangioma in the pancreatic body, as confirmed by postoperative histological examination and immunochemical testing.

## Case presentation

A 52-year-old female was admitted to our ward with a chief complaint of epigastric pain. The pain was described as intermittent unbearable, radiating to the back occasionally, and having no relationship to diet. There are no other symptoms associated with pancreatic lesion, such as weight loss, steatorrhea,diarrhea, nausea, acid regurgitation, or hematochezia. The physical examination of the abdomen was generally normal, except for mild tenderness over the epigastrium, without rebound tenderness.

The patient had a medical history of hypertension for 6 years and had been undergoing maintenance therapy with oral anti-hypertensive drugs. There was no history of abdominal operation or acute pancreatitis, and no history of alcohol intake or smoking.

Electrocardiography and chest radiography yielded normal results. The complete blood counts, serum electrolytes, glutamate pyruvate transaminase, aspartate aminotransferase, alkaline phosphatase, bilirubin, creatinine, amylase and glucose were in the normal range, but the serum total protein (62.1 g/l) and serum albumin (36.4 g/l) were slightly lower than normal. Tests for coagulation, serum CA 19–9, serum CA 24–2 and carcinoembryonic antigen (CEA) level were normal.

**Fig. 1:**
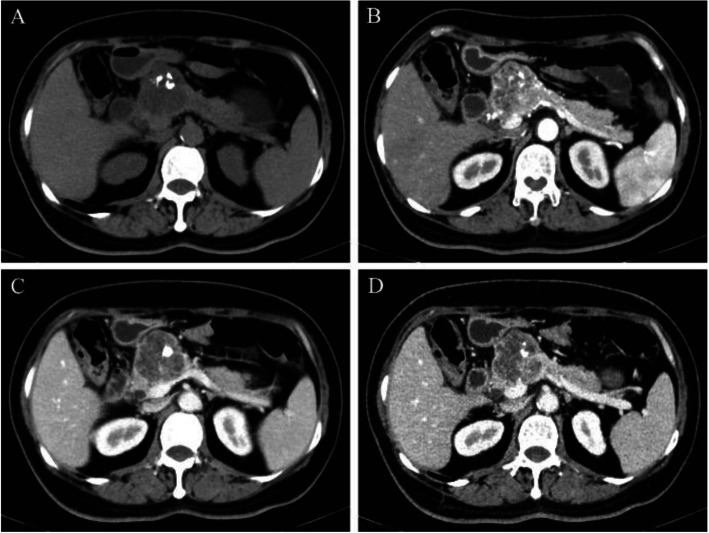
Dynamic CT scans of the pancreas showed a well-defined mass in the body of the pancreas. **a** Plain scan showed speckled and lamellar calcifications in the mass. **b** Arterial phase revealed mildly inhomogeneous enhancement. **c** Venous phase. **d** Delayed phase showed slight dilation of the main pancreatic duct

Abdominal computed tomography (CT) was performed after admission. A well-defined 46 mm × 46 mm × 34 mm mass with mildly inhomogeneous enhancement was confirmed in the body of the pancreas, along with speckled and lamellar calcifications (Fig. 1). The diameter of the common bile duct was normal, but the main pancreatic duct showed slight dilation. Endoscopic ultrasonography (EUS) showed a well-circumscribed mass with mixed echo in the body of the pancreas. The lesion contained calcifications showing strong echo. A few small vessels were detected by Doppler imaging. There was no ductal or vascular involvement. No peri-pancreatic lymph nodes were observed.

**Fig. 2:**
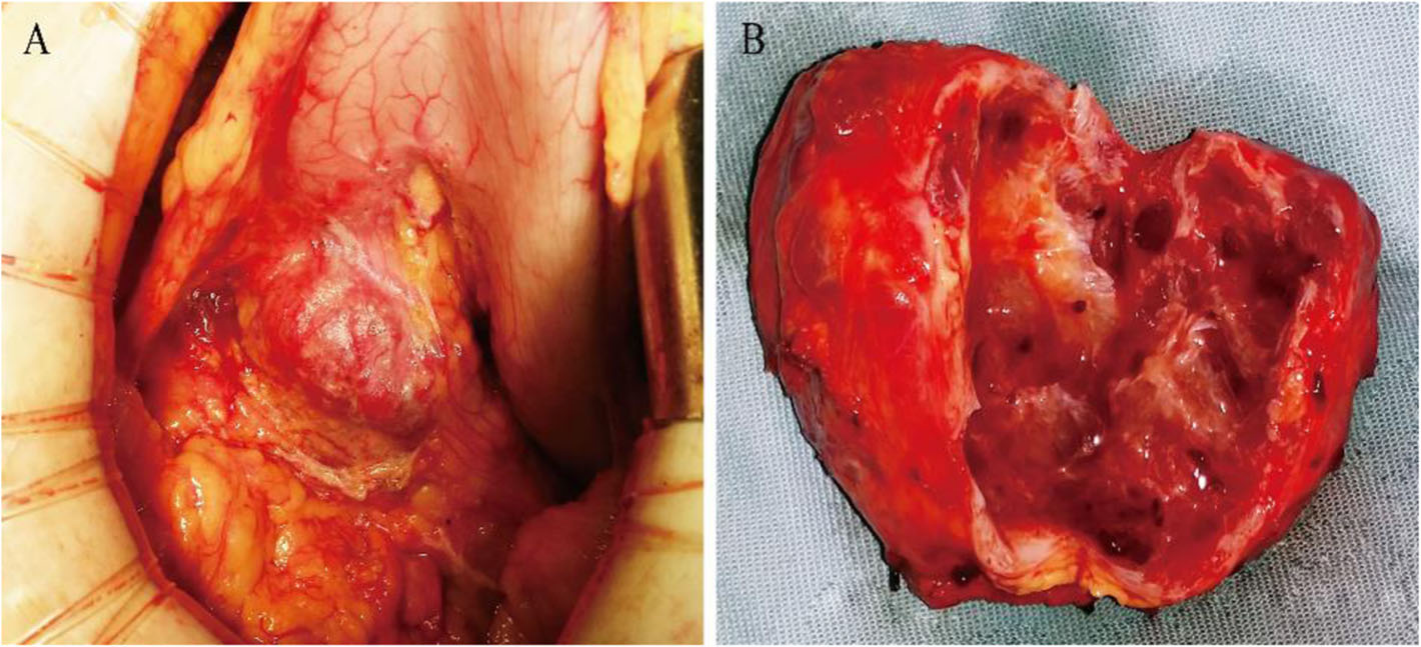
**a** The tumor was located in the pancreatic body and was adjacent to the gastric wall intraoperatively. **b** The gross pathologic specimen showed a 5.0 cm × 4.5 cm hemangioma containing multiloculated cysts

**Fig. 3:**
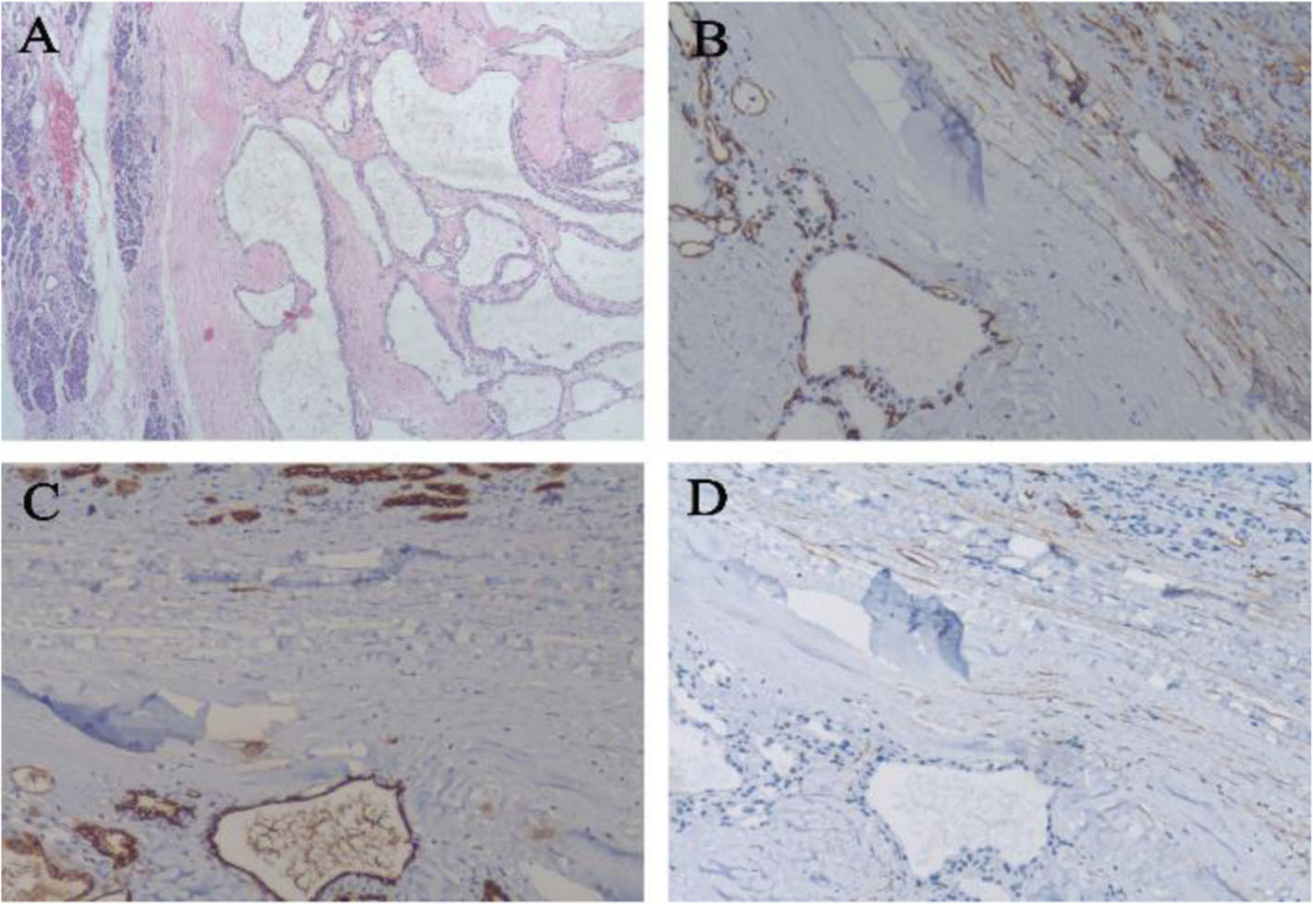
Histopathology. (**a**) H&E staining of the border of the tumor and normal pancreas (× 40 magnification). Strong positive CD34 staining (**b**) and positive CK staining (C) on the endothelial surfaces proved the vascular origin (× 100 magnification). Negative D2–40 staining excluded lymphangiomas (D, × 100 magnification)

Our preoperative diagnosis was pancreatic mass (mucinous cystic neoplasm?, serous cystic neoplasm?). Before the operation, we planned to perform either a pancreatoduodenectomy or possibly a central pancreatectomy depending on the intraoperative findings”. Eventually, we used a pancreaticojejunostomy to a Roux-en-Y limb; the pancreatic parenchyma was very soft, a tumor mass was found at the pancreatic body (Fig. 2), with dilated vessels on the surface of the mass. It was difficult to separate in the rear of the tumor, in front of the superior mesenteric vein and portal vein. But with careful dissection, a plane was developed and there was no involvement of the extrahepatic venous structures and no substantive bleeding. The gross pathologic examination revealed a 5.0 cm × 4.5 cm hemangioma mainly composed of multiloculated cysts with partly intracystic hemorrhage and no mucinous component (Fig. 2). In microscopic examination, the tumor showed a cavernous hemangioma composed of large vascular spaces lined by a single layer of uniform flattened cells, and no sign of malignancy was observed within the tumor (Fig. 3). In immunohistochemical staining revealed that the lining cells were positive for CD34 and CK, and negative for D2–40 (Fig. 3), thus supporting the diagnosis of hemangioma.

The postoperative course was uneventful, no pancreatic fistula developed, and the patient was discharged on the 7th postoperative day. The patient experienced no recurrence after 10 months of follow-up.

## Discussion and conclusions

Hemangiomas can be divided into infant and adult hemangiomas. Infant hemangiomas occur primarily in the skin and often gradually involute by 6–12 years of age, leaving a fibro-fatty residuum by adulthood [2]. Adult hemangiomas in the abdominal organs most frequently occur in the liver. Before now, fewer than 20 adult pancreatic hemangioma cases have been reported in the English literature since 1939 (Table 1).

**Table 1:** Related report of adult pancreatic hemangiomas in the English literature since 1939

No.	Year	Author	Age/sex	Symptoms	Diagnostic Imaging	Location	Size, cm	Preoperative diagnosis	Treatment
1	1939	Ranstrom [11]	61/F	Found at autopy	–	Head	7 × 7	Found at autopsy	–
2	1961	Ringoir et al. [12]	71/F	Hematemesis, melena	Abdominal plain film	Head	15	–	Retrocolic gastroenterostomy, vagotomy
3	1972	Colardyn et al. [13]	42/F	Abdominal/back pain	Abdominal plain film, angiography	Body/tail	–	–	Fat-free diet, anticholinergics
4	1991	Kobayashi et al. [4]	30/M	Abdominal distention	US, CT, MRI, angiography	Head	20	Cavernous hemangioma by MRI	PD
5	2003	Chang et al. [5]	70/F	Abdominal pain	US,CT, angiography	Body/tail	4 × 3.2 × 3	Serous/cystic adenoma/adenocarcinoma	Subtotal pancreatectomy
6	2006	Plank et al. [8]	36/M	Abdominal pain, jaundice	CT, MRI	Head	3	Nonfunctioning neuroendocrine tumor	Laparotomy
7	2009	Mundinger et al. [14]	45/F	Epigastric pain, radiating to back	US,CT, MRI	Head	6.2 × 5.3	Benign(duplication cyst, paraganglioma) cyst	PPPD
8	2011	Weidenfeld et al. [7]	73/F	Abdominal pain radiating to left flank	CT	Uncinate process	5.5 × 4 × 3	Cystic lesion with solid component	PD
9	2011	Lee et al. [15]	49/F	Dizziness, palpitation	US, CT, EUS	Neck	5	Mucinous cystic neoplasm	Central pancreatectomy, pancreaticogastrostomy
10	2013	Lu et al. [16]	23/F	No symptoms	US, CT, MRI	Head	5.4 × 5 × 3.1	Non enhancing multilocular cyst	Subtotal pancreatectomy
11	2013	Bursics et al. [9]	72/M	Epigastric pain, fever, jaundice	US, CT, ERCP	Head	8.7 × 7.8	Cystic tumor/IPMN	PPPD, pancreaticogastrostomy
12	2014	Naito et al. [10]	40/F	Abdominal pain	CT, MRI	Body/tail	10	Cystic neoplasm	Pancreatectomy
13	2014	Williamson et al. [17]	78/F	Epigastric pain	US, CT, EUS	Head/ uncinate process	4	Hemangioma by EUS	Observation
14	2015	Mondal et al. [1]	18/F	Epigastric pain, nausea, emesis	CT, MRI, EUS	Head	6.2 × 5.3	Benign cyst/neoplastic cystic lesion	PPPD, cholecystectomy
15	2015	Lu et al. [18]	28/F	Upper abdominal pain	CT	Body/tail	8.8 × 6.5 × 5.5	Cystadenoma/ pseudocyst	Subtotal pancreatectomy, splenectomy
16	2015	Søreide et al. [19]	30/F	Left upper quadrant abdominal pain	US, MRI	Tail	17 × 10	Solid pseudopapillary epithelial neoplasm	Distal pancreatectomy, splenectomy
17	2016	Kim et al. [3]	68/F	No symptoms	CT	Tail	0.6 × 0.5	Neuroendocrine tumor/renal cell carcinoma metastasis	Distal pancreatectomy
18	2016	Present case	52/F	Epigastric pain	CT, EUS	Body	4.6 × 3.4	Mucinous cystadenoma/ serous cystadenoma	Central pancreatectomy, pancreaticojejunostomy

*PD* Pancreatoduodenectomy; *PPPD* Pylorus preserving pancreatoduodenectomy; *US* Ultrasonography; *EUS* Endoscopic ultrasonography; *ERCP* Endoscopic retrograde cholangiopancreatography; *CT* Computed tomography; *MRI* Magnetic resonance imaging; *IPMN* Intraductal papillary mucinous neoplasm

A review of the cases in the English literature showed that most of the patients with adult pancreatic hemangioma were females (15/18), with an average age of 49 years (range: 18–78 years). The most common symptom was abdominal pain or epigastric pain (12/18). Other complaints included hematemesis and melena in one patient, abdominal distention in one patient, and dizziness and palpitations in another patient. One patient developed jaundice and fever, and another developed nausea and emesis in addition to the epigastric pain. Two patients had no obvious symptoms. In 10 patients, the tumors were located in the pancreatic head, and only two of these patients developed jaundice because of biliary compression by the tumors. In one patient, the tumor as located in the pancreatic neck, and in seven patients, it was located in the pancreatic body/tail. The hemangiomas were generally large in size, with an average diameter of 7.7 cm (0.6–20 cm). Patients with pancreatic hemangiomas were usually asymptomatic, or the symptoms were slight and not specific, which explains why the lesions were large in size. There was a patient who had a tumor with a diameter of 0.6 cm that was found during a medical checkup along with the presence of left renal cell carcinoma [3].

The diagnosis of adult pancreatic hemangioma is difficult preoperatively. Only two patients have been reported to be diagnosed with pancreatic hemangioma preoperatively. Cystadenoma, neuroendocrine tumors, cystic tumor/lesion/neoplasm, and intraductal papillary mucinous neoplasm (IPMN) are common preoperative diagnoses. CT is the optimal diagnostic option and was applied in 14 of the 18 patients. In most cases, the appearance of pancreatic hemangiomas on CT was different from that of conventional hemangiomas, such as liver hemangiomas. Liver hemangiomas typically show peripheral irregular enhancement first in the arterial phase, and then the entire tumor is filled in centripetally in the delayed phase. In contrast, adult pancreatic hemangiomas usually do not show significant arterial phase enhancement, possibly because of the cystic feature of pancreatic hemangiomas, which contain areas of neovascularization with arteriovenous shunting, and the blood flow through these cavernous vascular components is slow [4, 5]. Additionally, the ratio of the cystic component to solid tissue in the tumor determines the degree of tumor vascularity, which can affect the signal intensity on CT [6]. In our case, the tumor revealed mildly inhomogeneous enhancement but apparently lower intensity than normal pancreatic tissue, with small septa in the lesion. Therefore, it was first diagnosed as mucinous cystadenoma or serous cystadenoma. Speckled and lamellar calcifications were found on the plain scan of the present case. This has also been observed in one of the previous reports [7]. Magnetic resonance imaging (MRI) was performed in seven of the 18 patients. For non-enhanced MRI, pancreatic hemangioma often behaves with low signal attenuation on T1-weighted images and high signal attenuation on T2-weighted images [4]. In contrast, the tumors showed only moderate gadolinium-enhancement with washout on the delayed phase images, with no uptake of mangafodipir [8]. EUS is another method often used to diagnose adult pancreatic hemangioma, which was applied in four patients. The tumors were generally devoid of a obvious vascular flow on Doppler imaging [1]. Endoscopic ultrasonography-guided fine needle aspiration (EUS-FNA) or core biopsy provides an effective way to identify pancreatic hemangiomas preoperatively.

Pathologically, adult pancreatic hemangiomas generally show a typically cavernous hemangioma, as in our case, which is composed of cysts lined by a single layer of uniform endothelial cells. Tests using antibodies against CD31, CD34 or factor VIII-related antigen are used to confirm the vascular endothelial origin of the tumor. In the present case, the tumor was positive for CD34 and CK, but negative for D2–40, thereby supporting the diagnosis of hemangioma rather than other types of cystic neoplasm, particularly cystic lymphangiomas.

Because of the risk of bleeding, as well as the difficulty in differentiating them from other pancreatic epithelial tumors, most of the adult pancreatic hemangiomas received surgical resection. Operation was performed in 15 of the 18 patients diagnosed inpatients other than at autopsy. Out of these, 2 underwent, 2 underwent central pancreatectomy, 3 underwent pylorus preserving pancreatoduodenectomy, 3 underwent subtotal pancreatectomy, 2 underwent distal pancreatectomy, and 3 underwent other resections.

Pancreatic hemangiomas appear to grow very slowly and rarely cause obstruction of the common bile duct or invade the pancreatic duct, even when they are large. They have shown no malignant potential, so the need for aggressive surgical removal should be considered carefully. Owing to the difficulty of preoperative diagnosis, it is challenging for surgeons to make a decision. Percutaneous biopsy or endoscopic ultrasonography-guided fine needle aspiration (FNA) may be helpful in the diagnosis of pancreatic hemangioma. Additionally, the severity of the patients’ symptoms and the physical status of the patients must be considered during decision making. Because adult pancreatic hemangioma is a benign tumor, parenchyma-sparing pancreatectomy is a preferred surgical procedure, which can avoid the unnecessary resection of the normal pancreatic parenchyma, thereby maximally preserving the exocrine and endocrine functions of the pancreas. Local resection is a proper option for selected, peripheral, well defined capsulated lesions not close to the pancreatic duct without obvious parasitic vessels that could lead to uncontrolled bleeding, rather than pancreatoduodenectomy.

The texture of normal pancreatic parenchyma relative to pancreatic hemangioma is often soft, thus increasing the occurrence of postoperative pancreatic fistula. No severe postoperative pancreatic fistulas were reported in the 18 cases. The postoperative courses were uneventful in all cases except for a wound dehiscence that required secondary suturing in one patient [9]. Recurrence was not observed in any resected pancreatic hemangioma cases, including a patient without recurrence with 6 years of follow-up [10].

In conclusion, adult pancreatic hemangioma is a rare tumor type, and no specific clinical symptoms can be observed preoperatively. Correct preoperative diagnosis is difficult. CT is the most often used tool used to diagnose adult pancreatic hemangioma which is performed without a typically significant enhancement in the arterial phase. MRI can assist in diagnosis, and EUS-FNA is a suitable way to identify the content of the lesion. Owing to the benign potential of the pancreatic hemangioma, surgery should be considered carefully, and parenchyma-sparing pancreatectomy is recommended.

## Data Availability

The datasets used and/or analysed during the current study are available from the corresponding author on reasonable request.

## Abbreviations

CA19–9: Carbohydrateantigen19–9
CA24–2: Carbohydrate antigen24–2
CEA: Carcinoembryonic antigen
PD: Pancreatoduodenectomy
PPPD: Pylorus preserving pancreatoduodenectomy
US: Ultrasonography
EUS: Endoscopic ultrasonography
ERCP: Endoscopic retrograde cholangiopancreatography
CT: Computed tomography
MRI: Magnetic resonance imaging
IPMN: Intraductal papillary mucinous neoplasm.

## References

[1] Mondal U, Henkes N, Henkes D, Rosenkranz L (2015). Cavernous hemangioma of adult pancreas: a case report and literature review. World J Gastroenterol.

[2] Takahashi K, Mulliken JB, Kozakewich HP, Rogers RA, Folkman J, Ezekowitz RA (1994). Cellular markers that distinguish the phases of hemangioma during infancy and childhood. J Clin Invest.

[3] Kim SH, Kim JY, Choi JY, Choi YD, Kim KS. Incidental detection of pancreatic hemangioma mimicking a metastatic tumor of renal cell carcinoma. Korean J Hepatobiliary Pancreat Surg. 2016;20:93–96. . 10.14701/kjhbps.2016.20.2.93 PMC4874050 27212999

[4] Kobayashi H, Itoh T, Murata R, Tanabe M. Pancreatic cavernous hemangioma: CT, MRI, US, and angiography characteristics. Gastrointest Radiol. 1991;16:307–310. 10.1007/BF01887375 1936772

[5] Chang WT, Lee KT, Yang SF. Cavernous hemangioma of the pancreas: report of a case. Pancreas. 2003;26:310–312. 10.1097/00006676-200304000-00018 12657961

[6] Freeny PC, Weinstein CJ, Taft DA, Allen FH. Cystic neoplasms of the pancreas: new angiographic and ultrasonographic findings. AJR Am J Roentgenol. 1978;131(5):795–802. 10.2214/ajr.131.5.795 101030

[7] Weidenfeld J, Zakai BB, Faermann R, Barshack I, Aviel-Ronen S.Hemangioma of pancreas: a rare tumor of adulthood. Isr Med Assoc J. 2011;13:512–514. 21910381

[8] Plank C, Niederle B, Ba-Ssalamah A, Schimaa W. Pancreatic hemangioma: imaging features with contrast-enhanced CT and gadolinium and mangafodipir enhanced MRI. Eur J Radiol Ext. 2006;57:59–62.

[9] Bursics A, Gyokeres T, Bely M, Pörneczi B. Adult hemangioma of the pancreas: difficult diagnosis of a rare disease. Clin J Gastroenterol. 2013;6:338–343. 10.1007/s12328-013-0396-8 26181740

[10] Naito Y, Nishida N, Nakamura Y, Torii Y, Yoshikai H, Kawano H, Akiyama T, Sakai T, Taniwaki S, Tanaka M, Kuroda H, Higaki K. Adult pancreatic hemangioma: A case report. Oncology letters. 2014;8:642–644. 10.3892/ol.2014.2206 PMC4081133 25013478

[11] Ranstrom V. Haemangioma caverosum pancreatic. Zentralblatt für allgemeine pathologie und pathologische. 1939;73:33–35.

[12] Ringoir S, Derom F, Colle R, Mortier G. Hemangioma of the pancreas. Gastroenterology. 1961;41:43–45. 13741765

[13] Colardyn F, Elewaut A, Van de Velde E, Barbier F. Hemangioma of the pancreas. Tijdschr Gastroenterol. 1972;15:260–267. 4642101

[14] Mundinger GS, Gust S, Micchelli ST, Fishman EK, Hruban RH, Wolfgang CL. Adult pancreatic hemangioma: case report and literature review. Gastroenterol Res Pract. 2009;2009:839730. 10.1155/2009/839730 PMC2676326 19421421

[15] Lee J, Raman K, Sachithanandan S. Pancreatic hemangioma mimicking a malignant pancreatic cyst. Gastrointest Endosc. 2011;73:174-176. 10.1016/j.gie.2010.07.038 20932519

[16] Lu ZH, Wu M.Unusual features in an adult pancreatic hemangioma: CT and MRI demonstration. Korean J Radiol 2013;14:781–85. 10.3348/kjr.2013.14.5.781 PMC3772258 24043972

[17] Williamson JM, Finch-Jones M, Pope I. Endoscopic ultrasonography allowing expectant management of pancreatic haemangioma. Ann R Coll Surg Engl. 2014;96:e1-e2. 10.1308/003588414X13814021678231 PMC4474085 24780776

[18] Lu T, Yang C. Rare case of adult pancreatic hemangioma and review of the literature. World Journal of Gastroenterology. 2015;21(30):9228-9232. 10.3748/wjg.v21.i30.9228 PMC4533056 26290651

[19] Soreide JA, Greve OJ, Gudlaugsson E. Adult pancreatic hemangioma in pregnancy--concerns and considerations of a rare case[J]. BMC Surg, 2015,15:119. 10.1186/s12893-015-0106-1 PMC4628376 26518354

